# Medical Politics 101

**DOI:** 10.2349/biij.3.3.e13

**Published:** 2007-07-01

**Authors:** BJJ Abdullah, KH Ng

**Affiliations:** Department of Biomedical Imaging, Faculty of Medicine, University of Malaya, Kuala Lumpur, Malaysia

One of the penalties for refusing to participate in politics is that you end up being governed by your inferiors

–Plato (427 BC - 347 BC)

Today’s world faces problems that are complex and complicated. The field of medicine is showing rapid progress with new discoveries, policies and paradigms [1-4] and seeing new challenges which require new and rapid responses. Consequently, medicine and healthcare are being increasingly turning into a business [5].

Despite progress in many areas, there are certain key sectors within medicine that are chugging along rather slowly. We do not seem to have answers to medical politics and questions like:

- On what basis was the government acting with regard to issues of practice?
- Why is my treatment not covered by government or insurance?
- Why is the funding cut for specific procedures?
- Why are resources being allocated for something we don’t all agree upon?
- On what basis was the funds divided amongst all the different parties and how on earth did they get all that funding and space when they do not seem to have the required workload?
- How on earth did he get elected to that position over several more competent and credible candidates?

These are the all too familiar grouses heard amongst the public, physicians, ancillary staff and the administration. Everyone blames the politicians even though we really do not understand how the system works. Is there a better way to deal with controversial biomedical issues confronting us today? Can we anticipate the forces that will emerge on the various sides of an issue better, or are we destined to muddle through and institute policies incrementally and contentiously? [6]

Politics is often associated with dishonour and corruption because that is how it is frequently practised. The unfortunate truth is that political pressure has been imposed to alter scientific reports on everything from the environment to occupational health, and racial disparities in health care [7]. This is not as shocking as it seems. After all, we do manipulate in our daily lives and in politics, the level of manipulation is just a lot more.

As much as we would like to deny it, medicine is not immune to politics! Few of us in biomedicine want anything to do with politics because it is messy, chaotic and disordered, a far cry from the world we have been trained in. Politics, unlike medicine, is not evidence-based. Physicians and scientists believe they operate in a rational world, one in which interpretations and predictions are based on objective data and evaluated through a systematic process [6]. However, it would be a fallacy to assume they are without group-think and peer pressure.

Why the need for politics? The reason for all the jostling, manoeuvring and strategising is that there is never enough to go round for all the interested parties. The pie has to be divided and not everybody gets their share of it. While collective decision making is a solution, the desire to push individual agendas using every available means. This serves as to influence the decision making process and operates as political pressure. The decision-making can either occur with openness and honesty, or with subterfuge and dishonesty. In the latter case we are tempted to accuse: “Politics!” [8]. Sometimes the interests of all groups can be advanced although often, rigid deadlock occurs with little movement in any direction [6] no progress or benefit to any party. We therefore liken politics to a process by which a group reaches a decision [8].

Success in the political realm is no different from success in medicine, business or any other venture. Defining success may really be a question of looking at it in the context of time, place and circumstance. Therefore, a useful strategy for examining decision making is to separate the outcome from the quality of the decision process [6].

- Leaders must indeed create ideas and carry policies forward, but always consulting the led, creating buy-in and sense of being part of the process from those you lead. Followers may desire results with the least effort, sacrifice and contribution but maintain the right to complain and criticise when leadership is short on the delivery [9].
- Leaders are judged by how they spend their time, how they react to critical incidents, the stories they tell, the questions they ask, the language and symbols they choose and the measures they use [10].
- The operative word is 'networking.' If one wants to achieve lasting success in business, organised medicine or raising show dogs, one must build a network of people with similar interests" [11].
- You also need to be able to negotiate and sympathise with both sides of the argument and achieve an acceptable compromise. However, this should not be overdone and become Machiavellian, where political expediency is placed above morality, craft and deceit used to maintain authority and carry out the policies of a leader but rather ensuring that one’s skills are applied to principled purpose.
- Without your feet firmly on the ground and your shoulders able to hold your head, power and position can be addictive. Medical politics is as dangerous and habit-forming a drug as any benzodiazepine, and the sensible medical politician should have a level of self awareness to realise when participation has become self serving [9]. The position itself becoming the prized possession and no longer the possibility of bringing on change or the perceived good leadership is supposed to drive.
- Provide opportunity to those under your wing to grow and experience new learning. To quote Horace “no man ever reached to excellence in any one art or profession without having passed through the slow and painful process of study and preparation” [12]. As with most other pursuits, most of us learn by doing, and medical politics also requires an apprenticeship. The process from the beginning to the top may take decades as it may be intimidating, boring and even scary. Unless more of us take a turn in medical politics, our organisational life will wither, or worse, be left in the hands of political enthusiasts.
- Constantly “reinvent” yourself, staying updated on new developments, by learning from and responding to your environment. It is only by consciously breaking away from our own safe and comfortable paradigms and experimenting with new ones, from books, travelling, talking, arguing etc. can we bring a new perspective to our stories, add value to the lives of others and develop a different dimension. It is often a fear of failure that holds us back. But by stepping back a little and giving ourselves the time to think and analyse and learn, we avoid the rut.
- Do not to isolate yourself from your associates as you may end up being the one talked about, or blamed for miscellaneous, trivial problems. You also miss out on the real news in an organisation.
- Always ensure that one’s actions are based on the highest levels of moral practice, i.e. integrity, humility and leadership.

Success has a price, even though we may find it difficult to determine with certainty what the true costs are. What are all the costs associated with success in medical politics?

- Involvement in public life takes away some of your most valuable resources at our disposal, professional and creative time.
- Personal and professional practice is often strained because of competing priorities in a week.
- The toll is not only on yourself but our immediate families and friends, the organisations you work and in instances, the very patients you set out to protect. The emotional wear and tear on the individual with moments of anxiety, embarrassment, and rage tends to accumulate over time. It is not uncommon to see episodes of euphoria interspersed with longer periods of melancholy.
- When you finally decide to throw in the towel, you may find it hard to let go. Depression, anger and disgust are not uncommon and you may forget all the good things that have been done. This is especially true if the change-over has been filled with lots of turbulence, acrimony and “death to the end” battles. Sometimes you may even resent the successor, even if he or she is of your own choosing.
- If you lead or have been involved in many organisations, perhaps the best time to go is early on your prime, when you are still at your best. Set yourself a target of number of years or specific objectives and leave once that is achieved, with a good feeling. Do not wait until the politics of the organisation drive you out. Your supporters will always tell you are the best man for the job and that no one has your vision and your drive, but don’t fall for that! Most of them also carry vested interests and may be afraid of change.
- Rubbing shoulders with the powerful and influential adds to the sense of worth and purpose in being in the thick of regional, national and international decision making. Make sure you can handle it.

Even though Osler found health policy to be dull, eschewed political action committees as undignified and advised physicians to shun politics, there are those who believe that in today’s practice this laudable philosophy limits the health policy potential of the doctor-patient relationship [13]. The price of a physician’s closeness to his patients’ needs and experiences is to assume responsibility to look after their interests. An effective form of advocacy available to every physician is education: infusing health care policy into patient health care maintenance. Patients, who are voters, must be empowered to shape the local and federal policies that directly influence their healthcare.

In addition, there are those who go so far as to say that politics and management are obviously related. Management is the applied science; politics the high art. Political experience and training are the best introduction to management. The art of identifying what is possible and eliciting the best out of people is the basis of both [14]. One of these efforts is to frame policy issues as technical management questions [15-17] that are then best resolved by experts chosen based on merit. They hope to defang political conflict by appealing to evidence and expertise and search for a technocratic fix. The extension to this is that with evidence-based medical information, better clinical decisions, medical care, and health policies can be made without controversy or politics. And physicians, the public and governments will be able to rise above their parochial views and self-interest. However the nature of policy making is such that choices need to provide value and cannot be reduced to technical issues. It is not possible to purge issues of value, purpose, or politics from public policy. In fact, defining challenges in such a fashion masks the underlying political disputes. Battles over income, turf, and the goals of medicine and policy lie just below the surface. Under these circumstances, evidence can become an instrument of politics rather than a substitute for it [18].

Politics should be recognised, brought to the fore and included in training programmes for both medicine and research. The BMJ and The Lancet are trying to provide this by believing that serious medical journals should examine not only the immediate, but also the underlying causes of disease and premature death, which inevitably involve political issues [19-21]. To concentrate on the immediate causes, while ignoring the social and political factors underlying ill health, is in itself a political decision, after all “politics is nothing but medicine on a grand scale” [22]. It is also important to incorporate external political and human rights contexts into research ethics codes or ethics reviews. The balance of risks and benefits, the assurance of rights for individual participants, and the fair selection of research populations can be affected by the political and human rights background in which a study is done [23].

How do we go forward? Decision making is difficult when the members of the group do not trust each other, or feel secure. Politics can proceed in an atmosphere of trust, security, and knowledge, or without those benefits. In the former case, better decisions may be reached; they may not be perfect and they are unlikely to satisfy everyone, but they are not reached in an atmosphere of subterfuge and mistrust. And this itself may influence everyone concerned to surrender gracefully [8]. It would be exciting and gratifying to see some evidence-based politics in the health service sector [24].

A well lived “political” or “public” life has benefits for both the individual as well as to society at large. For the individual, it enhances reputation and respect, allows acquisition of the language and techniques of management, contributes to wider and better decision and policy making, the satisfaction of changing direction, focus, enhancing lives. It has been said that “A management course or two has become *de rigueur* for the sleek CV, but real organisational work is to management theory what making love is to a sex manual: both are interesting, but the practice is the more fulfilling experience” [25].
